# Nationwide registry-based analysis of rabies exposures in Iran: Epidemiological patterns and post-exposure prophylaxis adherence

**DOI:** 10.1371/journal.pntd.0014751

**Published:** 2026-09-30

**Authors:** Ebrahim Babaee, Arash Yousefzadeh Eshkoori, Babak Eshrati, Marzieh Nojomi, Mohammad Reza Shirzadi, Mehran Asadi-Aliabadi, Amirhesam Moosazadeh

**Affiliations:** 1 Preventive Medicine and Public Health Research Center, Psychosocial Health Research Institute, Department of Community and Family Medicine, School of Medicine, Iran University of Medical Sciences, Tehran, Iran; 2 Vaccine Research Center, Iran University of Medical Sciences, Tehran, Iran; 3 School of Medicine, Iran University of Medical Sciences, Tehran, Iran; 4 Department of Sociology & Anthropology, Nipissing University, North Bay, Ontario, Canada; 5 Zoonosis Control Department, Ministry of Health and Medical Education, Tehran, Iran; 6 Health Sciences Research Center, Mazandaran University of Medical Sciences, Sari, Iran; Washington State University, UNITED STATES OF AMERICA

## Abstract

**Background:**

Animal bites remain an important public health issue in Iran. Understanding their epidemiological patterns and examining how registry-recorded cases are managed in relation to the Iranian rabies protocol and World Health Organization recommendations can provide insight into clinical practice and documentation quality within the national bite registry. Such analyses help identify strengths and gaps in protocol adherence and data completeness.

**Methodology/Principal findings:**

We conducted a secondary analysis of 507,404 animal bite cases recorded in the national rabies registry between March 2022 and September 2023. The unit of analysis was registry-recorded exposure visits. Data on demographics, exposure characteristics, and clinical management were examined to describe epidemiological patterns and assess documented adherence to recommended post-exposure prophylaxis (PEP) practices. Males accounted for 73.6% of cases. The largest age group among all patients was 31–40 years, comprising 19.8% of cases. Dogs were responsible for 76.2% of exposures and cats for 20.8%. Northern provinces such as North Khorasan (773 per 100,000) and Golestan (767 per 100,000) recorded the highest incidence. Wound irrigation was documented in 94.3% of patients, and 96.4% received the first vaccine dose within 48 hours. Overall, 68.1% received three vaccine doses and 24.2% four doses. Rabies immunoglobulin was administered to 95.6% of Category III exposures, though inappropriate use was also observed in Category I (6.1%) and II (4.7%). Deviations from vaccine regimens and misclassification of exposure categories were identified, and among bat-related exposures only 36.8% were classified as Category III, with 38.7% receiving immunoglobulin.

**Conclusions/Significance:**

Among registry-recorded animal bite attendees, rapid initiation of PEP and frequent appropriate use of rabies immunoglobulin in severe exposures were documented. However, gaps in documentation, vaccine dosing accuracy, and exposure classification indicate areas for improvement. These findings reflect patterns within the national bite registry and should not be interpreted as a comprehensive evaluation of all surveillance system attributes. Strengthening provider training, reinforcing protocol adherence, and improving data quality within the registry could support national rabies control efforts and inform broader prevention strategies aligned with global elimination goals.

## Author summary

Rabies is a preventable disease, but it continues to pose risks in many parts of the world, including Iran. Animal bites are the main source of exposure, and understanding both their patterns and how they are managed is important for protecting communities. We analyzed over half a million reported bite cases across Iran to identify populations at highest risk and to evaluate adherence to recommended post-exposure prophylaxis practices. Our findings showed that most patients received timely wound care and vaccination, and almost all severe cases were treated with rabies immunoglobulin, which demonstrates the system’s ability to respond quickly. However, we also found some problems. In certain cases, wound washing was not recorded, vaccine schedules were not always followed, and some patients were classified into the wrong exposure category. For example, people exposed to bats were not always treated according to international guidelines. By highlighting both the strong points and the gaps, this study provides evidence that can help improve rabies prevention in Iran. Better training for healthcare workers, stricter adherence to protocols, and closer integration with measures such as dog vaccination and school-based education will further strengthen national efforts and bring the country closer to eliminating rabies.

## Introduction

Rabies is a zoonotic, neuroinvasive viral disease caused by the rabies virus (*Lyssavirus*, *Rhabdoviridae*), affecting all warm-blooded animals and targeting the central nervous system [1,2]. Transmission occurs mainly through the bite or saliva of infected animals, with wildlife such as raccoons, skunks, bats, and foxes serving as key reservoirs [1]. The virus persists in nature, reinforced by diverse and resilient wildlife reservoirs that sustain its circulation across ecological settings [3].

Once the rabies virus enters the body [4], it binds to nicotinic acetylcholine receptors at the neuromuscular junction and begins traveling along peripheral nerves toward the central nervous system (CNS) via retrograde axonal transport. This process allows the virus to evade the host’s immune response during its early stages, as it remains sequestered within neuronal pathways. The rabies virus replicates extensively upon reaching the CNS, causing neuronal dysfunction and the characteristic clinical manifestations of rabies [5].

Rabies progresses through distinct clinical stages, beginning with an incubation period that is highly variable - ranging from a few days to several years, but usually lasting one to three months - depending on factors such as bite location and viral load. This is followed by a prodromal phase characterized by nonspecific symptoms such as fever, malaise, and pain or paresthesia at the bite site [6–8]. The illness then advances to the acute neurologic stage, manifesting as either furious (encephalitic) rabies with agitation, hydrophobia, and aerophobia, or paralytic rabies, which occurs in roughly 20% of cases and presents with ascending weakness and paralysis [6,7,9]. Without intensive care, unvaccinated patients typically progress to coma and die within a few days. Once clinical symptoms appear, rabies is almost universally fatal [6–9].

Globally, rabies remains a significant public health issue, causing approximately 59,000 human deaths annually, with the majority of cases occurring in Africa and Asia [10]. Dogs are the source of infection in up to 99% of human rabies cases, and children between 5 and 14 years of age are the most commonly affected [11]; however, the specific animals responsible for transmission can vary by region. For instance, in the United States, while canine-specific rabies has been eradicated, the virus persists in wildlife such as bats, foxes, raccoons, and skunks [12]. In contrast, in Latin America and the Caribbean, human rabies cases have been linked to bats, dogs, and other species [1]. Although rabies is fatal once the clinical signs and symptoms appear, it is preventable if the post-exposure prophylaxis (PEP) starts immediately after contact with an infected animal [12].

Rabies surveillance systems have been widely advocated by global health organizations, particularly the World Health Organization (WHO) and the Centers for Disease Control and Prevention (CDC), due to their critical role in tracking and controlling this fatal zoonotic disease. According to WHO, effective surveillance provides essential data to inform vaccination strategies, monitor the disease burden, and evaluate the success of rabies control programs [13]. The CDC underscores that surveillance enables early detection of outbreaks and informs public health interventions, ultimately reducing human and animal cases [12]. Historically, robust systems in countries like the United States have nearly eradicated canine rabies through vaccination campaigns, while regions in Asia and Africa have made progress despite challenges such as underreporting and resource limitations. Strengthened surveillance has consistently reduced rabies cases and improved collaboration between public health and veterinary sectors [14].

Rabies remains a critical public health issue in Iran, with animal bites playing a major role in sustaining its transmission. In northern Iran, for example, an annual bite incidence rate of 386.3 per 100,000 people has been reported, with a consistently higher prevalence among males and a predominance of dog-related exposures [15]. National surveillance data further indicate that more than 260,000 animal bite cases occur annually (approximately 334 per 100,000 population), with domestic animals-particularly dogs-responsible for over 98% of reported bites and several human rabies deaths recorded each year despite the availability of PEP [16]. Despite the availability of PEP, rabies-related deaths continue to occur, underscoring the disease’s persistent mortality risk. The World Health Organization confirms the presence of dog-mediated human rabies in Iran, highlighting the ongoing transmission risk [17]. These findings emphasize the importance of addressing animal bites and implementing effective rabies control measures to reduce the disease’s burden.

Due to the significant public health burden of rabies and animal bites in Iran and in alignment with the WHO guidelines, Iran has established a national rabies surveillance system. However, few studies have examined the epidemiological characteristics and registry-recorded management of animal bite cases at the national level. Most existing research has focused on limited populations or specific aspects, such as the timeliness and initiation of PEP [16,18,19]. To address this gap, the present study provides a nationwide registry-based assessment of animal bite exposures and recorded rabies prophylaxis practices in Iran, with particular focus on epidemiological patterns, documentation completeness, timeliness of recorded care processes, and adherence to national and WHO-recommended PEP protocols. The primary research question examined whether the national rabies registry could characterize the epidemiological patterns of individuals presenting with animal bites and assess the consistency of recorded post-exposure prophylaxis practices with the Iranian national rabies guideline and WHO recommendations.

## Methods

### Ethics statement

Ethical approval Ethical approval for this study was obtained from the Ethics Committee of Iran University of Medical Sciences (Approval Code: IR.IUMS.REC.1403.088). All patient identifiers were removed prior to analysis to maintain confidentiality, and the study adhered to ethical principles for secondary data analysis.

This cross-sectional secondary analysis was conducted in Iran in 2024 using routinely collected data from the national rabies registry maintained by the Zoonoses Department of the Ministry of Health and Medical Education. The dataset included all registered cases of animal bites and rabies prophylaxis across the country during the Iranian year 1401 (March 2022-March 2023) and the first half of 1402 (February-September 2023), comprising a total of 507,404 cases. This study was based exclusively on registry-recorded bite and prophylaxis visits and was designed as a secondary analysis of routinely collected surveillance data. The unit of analysis was registry-recorded exposure visits. Because no external data sources or independent case ascertainment mechanisms were available, the analysis was restricted to epidemiological description, documentation completeness, and assessment of recorded adherence to PEP protocols. Broader surveillance system attributes such as sensitivity, representativeness, acceptability, stability, and predictive value could not be evaluated within the scope of this study. This study used all eligible registry records from the study period, so we did not perform a formal sample size calculation or sampling procedure.

In the national rabies registry, each record corresponds to a single individual for whom a medical file is opened at the time of first presentation following a rabies-related exposure or prophylaxis visit. Subsequent follow-up visits for additional vaccine doses or clinical reassessment are documented within the same unique identification number and do not generate new case entries. Accordingly, the unit of analysis in this study was the individual registry record rather than the number of clinic visits.

For the purposes of this analysis, operational definitions were applied to distinguish between different types of registry entries. An animal bite case was defined as any individual presenting with a documented bite exposure from a suspected or confirmed rabid animal. Non-bite exposure referred to individuals reporting mucosal contact, scratches without bleeding, or other indirect exposures to potentially rabid animals. Contact with an infected patient included individuals exposed to a confirmed human rabies case. Pre-exposure prophylaxis (PrEP) visits referred to individuals receiving vaccination in the absence of a documented exposure event. A registry record was defined as a unique individual-level entry in the national rabies database corresponding to a single exposure episode or initiation of prophylaxis. Incidence calculations were restricted to documented animal bite cases, while other visit reasons were described separately to prevent inflation of exposure-based estimates and to maintain consistency in denominators across analyses.

Extracted variables were categorized into several domains and analyzed accordingly. Demographic characteristics including age, sex, occupation, and place of residence (urban or rural) were used to describe the epidemiological profile of bite victims and to identify subgroups at higher risk. Exposure-related factors such as type of animal, vaccination status of the animal, number of wounds, and anatomical site of injury were analyzed to determine the major sources of rabies transmission and the clinical severity of exposures. Clinical management indicators including wound irrigation, antiseptic use, bandaging, wound closure, antibiotic administration, patient classification according to the national guideline (Categories I-III), number and route of vaccine doses (intradermal or intramuscular), volume of day 0 dose, and administration and timing of rabies immunoglobulin (RIG) were assessed to examine documented clinical management practices and adherence to national and WHO PEP protocols within the national rabies registry [20]. Exposure categories, wound management procedures (including wound irrigation and antiseptic application), and occupational classifications are defined in detail in Supplementary Tables S1–S4 Tables [20,21].

PEP doses and routes were recorded according to the Iranian national rabies guideline and WHO recommendations. The intramuscular schedule consists of four 1.0 ml doses administered on days 0, 3, 7, and 14, whereas the intradermal schedule consists of three 0.1 ml doses per site (0.2 ml total) on the same schedule. In our analysis, we report the doses and routes exactly as recorded in the registry, focusing on deviations from these recommended regimens.

Data completeness was assessed for key analytical variables. Some variables, such as antibody titers and later vaccine doses, were applicable only to specific clinical subgroups; therefore, missing values in these fields largely reflect protocol-based non-applicability rather than data recording errors. The proportion of missing values was calculated for each variable and is presented in S4 Table. Percentages reported in tables were calculated using available-case denominators unless otherwise specified.

In accordance with WHO guidelines, PEP should be administered as soon as possible after exposure. In the Iranian national rabies surveillance system, vaccine initiation is categorized into predefined time intervals, with the first dose administered within 48 hours considered timely, and subsequent doses recorded in intervals of <48 h, 48–72 h, 4–10 days, 11–20 days, 20–40 days, and >40 days. RIG is classified as timely if given within 12 hours, with delayed administration up to 72 hours considered acceptable when immediate infiltration is not possible. Using these operational categories, we evaluated how promptly patients received each vaccine dose and RIG, highlighting that shorter delays represent better adherence to timely PEP.

Vaccination history and reasons for prophylaxis (animal bite, non-bite exposure, contact with rabies patients, or pre-exposure prophylaxis) were analyzed to differentiate between post-exposure and pre-exposure interventions, while specific exposures such as bat-related cases were examined separately to assess correct classification and management. Descriptive statistics including frequencies, percentages, and incidence rates per 100,000 population were used to summarize the characteristics of exposures and interventions, and subgroup analyses were conducted to evaluate variations across demographic and occupational groups. Comparative analyses of patient management across exposure categories (I, II, and III) allowed assessment of alignment with recommended guidelines. Geographic patterns of animal bite incidence were examined at the provincial level using ArcGIS software (version 10.8.2; ESRI, Redlands, CA, USA). Provincial incidence rates were calculated by dividing the number of documented animal bite cases by the corresponding provincial population obtained from the most recent national census and official population estimates. For the Iranian year 1401 (March 2022–March 2023), incidence represents a full 12-month annual rate. For February–September 2023 (first half of Iranian year 1402), incidence represents period incidence for the reported months and was calculated using the full 2023 provincial population as the denominator. These rates were not annualised; therefore, comparisons between the two periods should be interpreted with caution.

Prior to analysis, data were reviewed for duplicate identifiers, and no duplicate records were identified. Basic data-cleaning procedures were performed, including range checks for continuous variables (e.g., age), verification of internal logical consistency across related variables (e.g., antibody titers recorded only among vaccinated animals), and validation of categorical coding. Implausible or inconsistent entries were examined and corrected where possible based on registry documentation. Analyses were conducted using available-case denominators for each variable. Since the study used all eligible registry records from the study period instead of a sample, the analyses focused on describing the data. Inferential statistical tests were not used because there was no sampling-based uncertainty in this nationwide census of registry records. Statistical analyses were performed with Stata software (version 17; StataCorp, College Station, TX, USA).

## Results

The study included 507,404 registry records of people who sought care for rabies-related exposure or prevention between March 2022 and September 2023. Of these, 63.07% (320,025) were recorded during the Iranian year 1401 (March 2022 to March 2023), and 36.93% (187,379) during the first half of 1402 (February to September 2023). Most records, 498,793 (98.30%), were for documented animal bites. The rest involved non-bite exposures, contact with infected patients, or pre-exposure prophylaxis (PrEP) visits. Unless noted otherwise, descriptive analyses used all registry records, while incidence estimates focused only on animal bite cases. There were few missing values for animal species (0.97%). More missing data appeared for animal vaccination history and antibody titers, mainly because these details were only collected for certain animals that had follow-up or lab testing. The timing of later vaccine doses followed standard protocols. Males made up 73.64% of all records. Table 1 shows that the largest age groups were 31–40 years (about 20%), 21–30 years (18%), and 11–20 years (17%), followed by children aged 0–10 years (14%).

**Table 1:** Descriptive analysis of the study population.

Variable	Categories	Number (%)
Year of bite	March 2022 - March 2023 (Iranian year 1401)	320025 (63.07)
	February 2023 - September 2023 (first half of Iranian year 1402)	187379 (36.93)
Sex	Male	373646 (73.64)
	Female	133758 (26.36)
Occupation	Healthcare staff	3336 (0.66)
	Firefighters	1166 (0.23)
	Livestock farmers	34518 (6.80)
	People working with non-domestic animals	1433 (0.28)
	Veterinarians	3890(0.77)
	Students	109357 (21.55)
	Children	32303 (6.37)
	Others	321401 (63.34)
Region	Urban	296419 (64.57)
	Rural	162640 (35.43)
Age group	0-10	71235 (14.06)
	11-20	85327 (16.84)
	21-30	90124 (17.79)
	31-40	100056 (19.75)
	41-50	68091 (13.44)
	51-60	48800 (9.63)
	61-70	29390 (5.80)
	71-80	10325 (2.04)
	80 <	3358 (0.66)
Reason for vaccination	Animal bite	498793 (98.30)
	Non-bite contact with suspected animal	3584 (0.71)
	Contact with infected patient	1022 (0.20)
	Pre-exposure prophylaxis	4005 (0.79)
Vaccination history before exposure	Non-vaccinated	475285 (93.67)
	Vaccinated	32119 (6.33)
Time passed since previous vaccination	Less than 3 months	411 (1.28)
	More than 3 months	31708 (98.72)

Occupationally, most cases belonged to other occupations (63.3%, n = 321,401), followed by students (21.6%, n = 109,357), livestock farmers (6.8%, n = 34,518), and children (6.4%, n = 32,303). High-risk occupations accounted for a smaller proportion of cases, including veterinarians (0.8%, n = 3,890), people working with non-domestic animals (0.3%, n = 1,433), and firefighters (0.2%, n = 1,166).The primary reason for vaccination was animal bites, accounting for 98.30% (n = 498,793) of cases. Other reasons included non-bite contact with rabies-suspected animals (0.71%, n = 3,584), contact with infected patients (0.20%, n = 1,022), and PrEP (0.79%, n = 4,005). Prior vaccination was reported by 6.33% (n = 32,119) of patients, and almost all (98.72%) had received their last dose more than three months earlier (**Table 1**).

Geographically, the distribution of cases across Iran showed higher incidence in the northern provinces as well as in central regions, as illustrated in Figs 1 and 2. In March 2022 - March 2023 (Iranian year 1401), the highest incidence rates were observed in North Khorasan (773 per 100,000), Golestan (767), and Mazandaran (668). Central and western provinces such as Chaharmahal and Bakhtiari (554), Gilan (537), Hamedan (499), and Ardabil (484) also showed elevated rates. During February - September 2023 (first half of the Iranian year 1402), a similar spatial pattern persisted, with the highest incidence reported in Mazandaran (413 per 100,000), followed by North Khorasan (400), Golestan (383), and Gilan (354). Central provinces such as Chaharmahal and Bakhtiari (307) and Lorestan (257) also had elevated rates.

**Fig 1:**
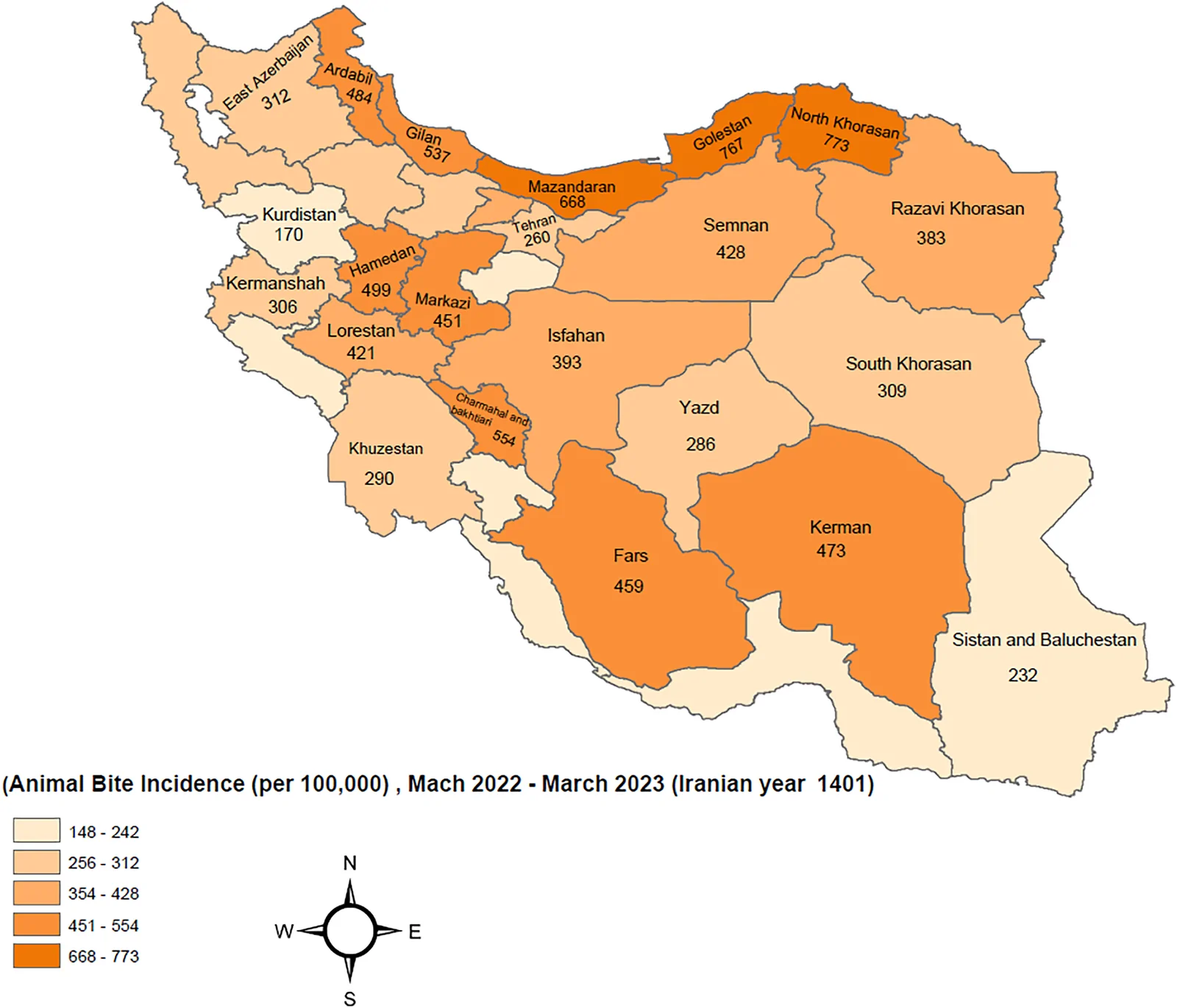
Geographic distribution of animal bite incidence across Iran during March 2022–March 2023 (Iranian year 1401). Provinces are shaded according to annual incidence per 100,000 population, calculated by dividing the total number of registered animal bite cases during the 12-month period by the corresponding provincial population obtained from the most recent national census and official population estimates. Darker colors indicate higher incidence rates. The basemap used in this figure is sourced from Natural Earth, which provides free public-domain vector and raster map data (naturalearthdata.com).

**Fig 2:**
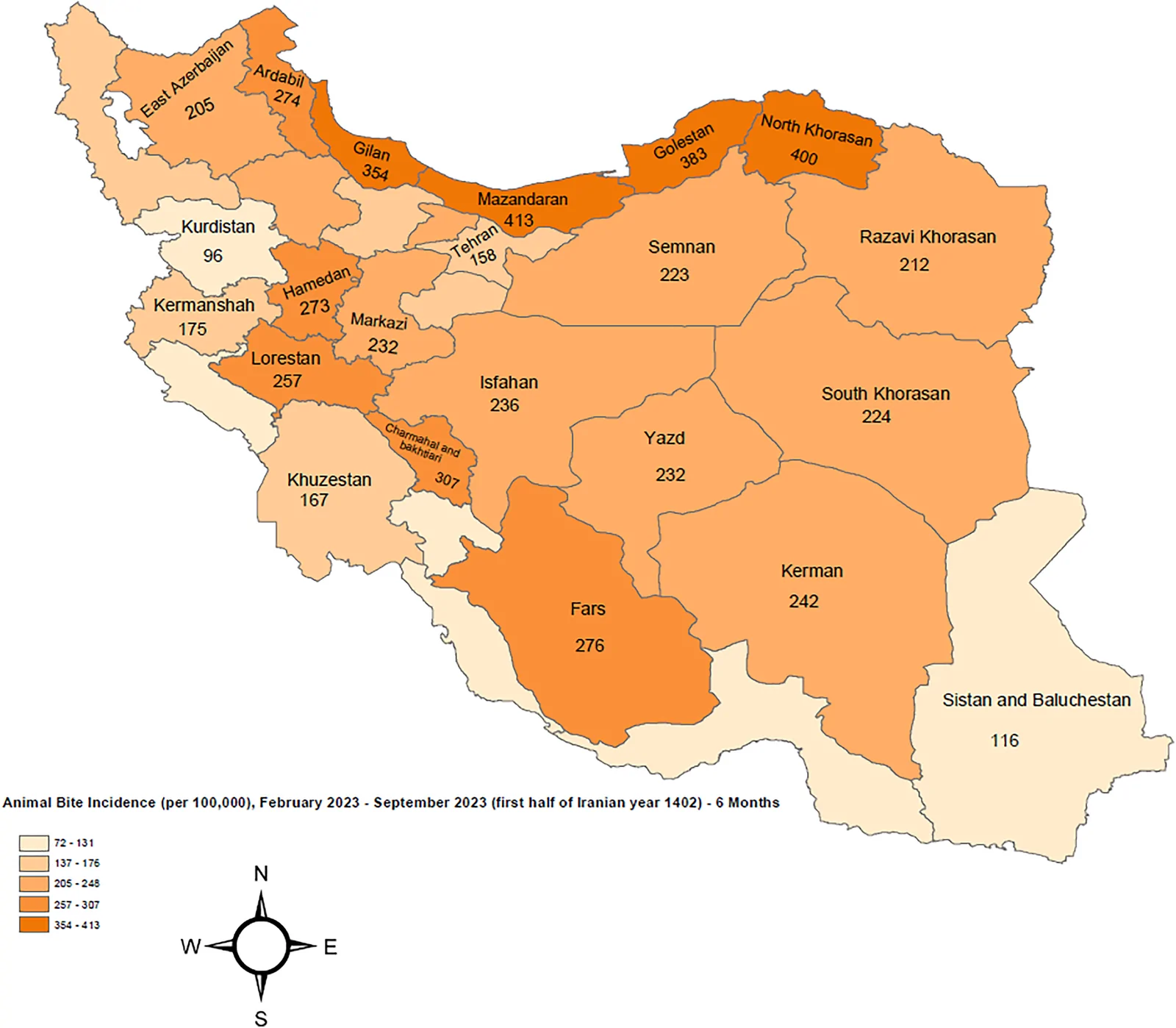
Geographic distribution of animal bite incidence across Iran during February–September 2023 (Iranian year 1402, first half). Provinces are shaded according to period incidence per 100,000 population, calculated by dividing the number of registered animal bite cases during the reported months by the corresponding 2023 provincial population estimates. These rates represent period incidence for the specified months and were not annualised; therefore, direct comparison with the 12-month rates shown in Fig 1 should be interpreted with caution. Darker colors indicate higher incidence rates. The basemap used in this figure is sourced from Natural Earth, which provides free public-domain vector and raster map data (naturalearthdata.com).

Regarding wound characteristics and the animals responsible, dogs accounted for the majority of exposures (76.15%, n = 382,627), followed by cats (20.79%, n = 104,488), whereas other animal species collectively represented only 3.06% of cases (**Table 2**). Among animals with available vaccination records, most were non-vaccinated (70.08%). Antibody titers were available for only 185 vaccinated animals and were almost equally distributed above and below the 0.5 IU/mL threshold. Nearly half of the patients sustained a single wound (48.97%), and bites most frequently involved the lower limbs (42.08%) and hands (39.73%), while injuries to other anatomical sites were much less common (**Table 2**). Detailed information on wound number, bite location, and animal-related characteristics is presented in **Table 2**. Percentages were calculated based on available data for each variable because of missing information in some records.

**Table 2:** Information regarding the wounds and responsible animals.

Variable	Categories	Number (%)
Animal	Dog	382627 (76.15)
	Cat	104488 (20.79)
	Bat	106 (0.02)
	Wild	3155 (0.63)
	Livestock	6057 (1.21)
	Rodent	5128 (1.02)
	Monkey	919 (0.18)
Animal vaccination history	Non-vaccinated	102426 (70.08)
	Vaccinated	43730 (29.92)
Antibody titers in vaccinated animals	Less than 0.5 UI/ml	96 (51.89)
	More than 0.5 UI/ml	89 (48.11)
	Total	185 (100.00)
Number of the wounds	One	229841 (48.97)
	Two	138741 (29.56)
	Three	53777 (11.46)
	More than three wounds	47020 (10.02)
Head and Neck bites	No	489831 (96.54)
	Yes	17573 (3.46)
Face and mucosal surfaces bites	No	505663 (99.66)
	Yes	1741 (0.34)
Bites in the Trunk	No	490301 (96.63)
	Yes	17103 (3.37)
Forearm and Shoulder bites	No	445417 (87.78)
	Yes	61987 (12.22)
Bites in the Hands	No	305836 (60.27)
	Yes	201568 (39.73)
Lower limb bites	No	293876 (57.92)
	Yes	213528 (42.08)
Genitalia bites	No	506673 (99.86)
	Yes	731 (0.14)
Ano-genital mucosal surfaces bites	No	507276 (99.97)
	Yes	128 (0.03)

Medical management showed that wound irrigation was performed in most cases (94.26%, n = 478,299), followed by antiseptic administration (76.96%, n = 390,506), whereas bandaging (0.31%), wound closure (0.03%), and antibiotic administration (17.71%) were considerably less frequent (Table 3). According to the national rabies guideline, most patients were classified as Category II (62.25%, n = 312,929), followed by Category III (36.25%, n = 182,230), while only 1.50% (n = 7,541) were classified as Category I. Regarding post-exposure prophylaxis, most patients received the recommended three-dose vaccination regimen (68.14%, n = 344,333), followed by four doses (24.19%, n = 122,225), whereas only a small proportion received two doses (7.57%) or no vaccination (0.10%). RIG was administered to 37.22% (n = 188,814) of patients, with the majority (81.15%) receiving it within 12 hours of exposure (Table 3). Adherence to the vaccination schedule was high, with more than 95% of patients receiving each scheduled vaccine dose within 48 hours. Detailed information on vaccine administration delays for each dose is presented in **Table 3**.

**Table 3:** Descriptive analysis of the interventions in medical centers.

Variable	Categories	Number (%)
Wound irrigation	Yes	478299 (94.26)
	No	29105 (5.74)
Antiseptic administration	Yes	390506 (76.96)
	No	116898 (23.04)
Bandaging	Yes	1595 (0.31)
	No	505809 (99.69)
Wound closure	Yes	144 (0.03)
	No	507260 (99.97)
Antibiotic administration	Yes	89859 (17.71)
	No	417545 (82.29)
Classification of patients based on protocol	Category I	7541 (1.50)
	Category II	312929 (62.25)
	Category III	182230 (36.25)
Number of administered vaccine doses	None	507 (0.10)
	Two doses	38253 (7.57)
	Three doses	344333 (68.14)
	Four doses	122225 (24.19)
RIG administration	Yes	188814 (37.22)
	No	318529 (62.78)
RIG administration timing	Less than 12 h	153225 (81.15)
	Between 12 h-72 h	32573 (17.25)
	More than 4 days	3016 (1.60)
Delay in day 0 vaccine administration	Less than 48 h	486478 (96.37)
	48-72 h	10986 (2.18)
	4-10 days	6209 (1.23)
	11-20 days	780 (0.15)
	20-40 days	216 (0.04)
	More than 40 days	142 (0.03)
Delay in day 3 vaccine administration	Less than 48 h	390636 (96.40)
	48-72 h	9281 (2.29)
	4-10 days	4430 (1.09)
	11-20 days	614 (0.15)
	20-40 days	184 (0.05)
	More than 40 days	84 (0.02)
Delay in day 7 vaccine administration	Less than 48 h	326370 (95.50)
	48-72 h	7802 (2.28)
	4-10 days	6492 (1.90)
	11-20 days	766 (0.22)
	20-40 days	214 (0.6)
	More than 40 days	116 (0.3)
Delay in day 14 vaccine administration	Less than 48 h	32684 (95.66)
	48-72 h	613 (1.79)
	4-10 days	451 (1.32)
	11-20 days	386 (1.13)
	20-40 days	28 (0.08)
	More than 40 days	4 (0.01)

Among the 507,404 patients assessed, 6.33% (n = 32,119) reported a prior history of rabies vaccination. Vaccination rates varied by occupation, with the highest rate observed among individuals working in animal-related jobs, including veterinarians (29.65%, n = 1,153 out of 3,890) and people working with non-domestic animals (10.46%, n = 150 out of 1,433). Other notable groups included firefighters (9.61%, n = 112 out of 1,166), healthcare staff (8.09%, n = 270 out of 3,336), and livestock farmers (8.62%, n = 2,977 out of 34,518). Students and children demonstrated lower rates at 4.84% (n = 5,289 out of 109,357) and 2.01% (n = 648 out of 32,303), respectively.Based on patients’ self‑reported information, 35.34% (n = 30,145) of 85,292 biting dogs were vaccinated against rabies. Reported vaccination rates varied by dog type, with hunter dogs having the highest rate (59.76%, n = 147/246), followed by pet dogs (47.68%, n = 18,086/37,935), guard dogs (27.01%, n = 10,480/38,805), and shepherd dogs (17.24%, n = 1,432/8,306).

The distribution of post-exposure interventions across different types of suspected animal exposures, classified according to the national rabies guideline (Categories I, II, and III), is summarized in Table 4. Across all exposure categories, the three-dose vaccination regimen was the most frequently administered, accounting for 69.97% of Category I, 70.51% of Category II, and 65.61% of Category III cases. Four-dose vaccination was more common in Category III (30.42%) than in Categories II (20.82%) and I (15.56%) (Table 4). RIG administration differed markedly across exposure categories. It was administered to only 6.13% of Category I and 4.66% of Category II cases but to 95.58% of Category III cases. Wound irrigation was performed in most patients regardless of exposure category, ranging from 86.48% in Category I to over 94% in Categories II and III (Table 4).

**Table 4:** Frequency distribution of Post-Exposure Interventions (Vaccination, RIG Administration, and Wound Irrigation) by Type of Exposure to Suspected Rabid Animal according to the National Rabies Guideline Classification (Categories I, II, and III).

Variable	Classification of patients			
		I	II	III
Number of administered vaccine doses	None	334 (4.98)	115 (0.04)	37 (0.02)
	Two doses	635 (9.48)	26712 (8.63)	7133 (3.95)
	Three doses	4689 (69.97)	218194 (70.51)	118515 (65.61)
	Four doses	1046 (15.56)	64418 (20.82)	54949 (30.42)
RIG administration	Yes	411 (6.13)	14473 (4.66)	173450 (95.58)
	No	6289 (93.87)	296095 (95.34)	8020 (4.42)
Wound irrigation	Yes	5795 (86.48)	299310 (96.37)	171164 (94.30)
	No	906 (13.52)	11275 (3.63)	10343 (5.70)

Among the 4,005 individuals whose reason for visit was recorded as PrEP, 270 were classified into Categories I–III according to the national rabies guideline. Of these, most were classified as Category II (77.0%, n = 208), followed by Category III (20.4%, n = 55), while only 2.6% (n = 7) were classified as Category I. Regarding vaccination, the majority of individuals received two doses (82.4%, n = 3,272), whereas 14.4% (n = 572) and 3.1% (n = 124) received three and four doses, respectively. RIG administration and wound management interventions were uncommon in this group. Detailed distributions of vaccination, RIG, and wound management are presented in Table 5.

**Table 5:** Distribution of Post-Exposure Interventions (vaccine doses, RIG, antiseptic, antibiotic, and wound irrigation) among individuals presenting for pre-exposure prophylaxis, categorized by.

Variable		Number (%)
Exposure Category	I	55 (20.37)
	II	208 (77.04)
	III	7 (2.59)
Number of administered vaccine doses	None	2 (0.05)
	Two doses	3272 (82.42)
	Three doses	572 (14.41)
	Four doses	124 (3.12)
RIG administration	Yes	4 (0.1)
	No	3996 (99.9)
Wound irrigation	Yes	31 (0.77)
	No	3974 (99.23)
Antibiotic administration	Yes	9 (0.22)
	No	3996 (99.78)
Antiseptic administration	Yes	18 (0.45)
	No	3987 (99.55)

The distribution of administered vaccine doses showed that 91.14% (n = 233,738) of intradermal recipients received three doses, 7.76% (n = 19,890) received two doses, and 1.10% (n = 2,827) received four doses. In comparison, 48.08% (n = 119,398) of intramuscular recipients received four doses, 44.53% (n = 110,595) received three doses, and 7.39% (n = 18,363) received two doses. The vaccine dose volumes on day 0 were recorded as 0.2 ml in the majority of intradermal recipients (96.12%, n = 246,509), followed by 0.1 ml in 2.49% (n = 6,383) and 1 ml in 1.39% (n = 3,563). By contrast, all intramuscular recipients (100%, n = 248,356) received 1 ml as the day 0 dose (Table 6).

**Table 6:** Distribution of Intramuscular and Intradermal rabies vaccination: number of doses administered and day 0 dose volume.

Variable		Intradermal	Intramuscular
Number of administered vaccine doses	Two doses	19890 (7.76)	18363 (7.39)
	Three doses	233738 (91.14)	110595 (44.53)
	Four doses	2827 (1.1)	119398 (48.08)
Vaccine dose volume on day 0	0.1 ml	6383 (2.49)	0
	0.2 ml	246509 (96.12)	0
	1 ml	3563 (1.39)	248356 (100)

Among the 106 individuals exposed to or injured by bats, most were classified as Category II (62.26%, n = 66), followed by Category III (36.79%, n = 39), while only one case (0.94%) was classified as Category I. All exposed individuals received rabies vaccination; overall, 57.55% (n = 61) received four doses, 33.96% (n = 36) received three doses, and 8.49% (n = 9) received two doses. RIG was administered to 38.68% (n = 41) of cases. Detailed information on vaccination by exposure category is presented in Table 7.

**Table 7:** Exposure category, number of administered vaccine doses, and RIG administration in individuals exposed or bitten by bats.

Variable		Number (%)
Exposure Category	I	1 (0.94)
	II	66 (62.26)
	III	39 (36.79)
Number of administered vaccine doses	None	0
	Two doses	9 (8.49)
	Three doses	36 (33.96)
	Four doses	61 (57.55)
RIG administration	Yes	41 (38.68)
	No	65 (61.32)

## Discussion

This nationwide registry-based analysis examined more than half a million animal bite and prophylaxis records documented between March 2022 and September 2023 (corresponding to 1401 and the first half of 1402 in the Iranian calendar). By analyzing registry-recorded data, the study provides insight into the epidemiological profile of animal bites in Iran, documented clinical management practices, and adherence to Iran’s National Rabies Control and Prevention Guideline [21], in comparison with WHO recommendations [11]. While the findings offer important information regarding patterns of exposure and protocol adherence among recorded attendees, the analysis was limited to routinely collected registry data and does not constitute a comprehensive evaluation of all surveillance system attributes. Instead, the results should be interpreted as a registry-based assessment of documented management practices and data completeness within the national reporting system.

Our national dataset revealed a male predominance among animal bite victims (73.6%). This is consistent with a national registry in 2021–2022 (77%) [16] and regional studies from East Azerbaijan (85%) [22], Semirom in Isfahan province (76%) [23], and Gilan (65.9%) [15], confirming that men are consistently more affected. The literature attributes this pattern largely to greater occupational and outdoor exposure, with men in these regions more frequently engaged in activities that increase contact with potentially rabid animals [22,23]. Additional contributing factors described include a higher concentration of rural residence among male victims, a cultural tendency among males toward greater daring in interactions with animals [22], and age‑related patterns, with men more often being very young or elderly compared to female victims [15]. Collectively, these findings indicate that sex differences in bite incidence are closely linked to behavioral, occupational, and exposure‑related factors documented across national and regional studies. In terms of age, the highest burden in our study was observed in adults aged 31–40 years, in line with data from East Azerbaijan (mean 31.8 years) [22]. In contrast, Semirom reported adolescents (10–19 years) [23], while Gilan found older adults (>50 years) were the most affected group [15]. These variations likely reflect demographic, occupational, and lifestyle differences across provinces. Overall, national and regional evidence highlight men and younger adults as the main risk groups for animal bites in Iran, while regional heterogeneity underscores the need for context-specific preventive strategies.

Our findings showed that students (21.55%) and children (14.06%) constitute a large proportion of bite victims. A meta-analysis of 33 Iranian studies similarly reported that students accounted for 20.1% of cases [24], highlighting the consistent vulnerability of this group and suggesting gaps in program implementation. This aligns with WHO recommendations for community and school-based rabies education [11]. International evidence also supports this approach. Studies in Bhutan revealed persistent knowledge gaps among school children [25], while curriculum integration in the Philippines significantly improved knowledge and reduced bite incidence [26]. Similarly, in Malawi, even a single classroom lesson produced sustained improvements in children’s knowledge and attitudes [27]. Taken together, these findings underscore the importance of structured, school-based rabies education programs in Iran.

Geographically, data indicate higher animal bite incidence in northern and western provinces, with Golestan, North Khorasan, Mazandaran, and Ardabil leading among northern provinces, Hamedan, Markazi, Lorestan, and Chaharmahal and Bakhtiari leading among western provinces, and Kerman and Fars having the highest incidence rates among southern provinces. Regional differences in the number of care-seekers may be explained by multiple factors: variations in occupational exposure (e.g., farmers, shepherds, veterinarians), frequency of contact with rabies reservoirs and densities of free‑roaming dogs, as well as disparities in access to PEP clinics. Lower reported cases from some southern provinces might partly reflect reduced medical care‑seeking due to limited public awareness, combined with local differences in wildlife populations, climatic conditions, and socioeconomic and lifestyle factors.

Urban areas accounted for 64.57% of bites in our study. This finding is consistent with a population-based study in Tehran, which reported that 66.3% of cases occurred in urban settings [19]. However, it contrasts with other Iranian evidence. For instance, a recent epidemiological study from Iranshahr in southeastern Iran found a rural predominance, with 67.1% of cases [28]. Similarly, a national meta-analysis of 33 studies reported nearly equal urban and rural distributions (49.7% rural) [29]. These discrepancies suggest that urban-rural differences may vary considerably by region and study period. The variation is likely influenced by heterogeneity in stray dog populations, human population density, health-seeking behavior, and access to post-exposure prophylaxis. Moreover, the increasing trend of pet ownership, particularly of dogs, in urban households may also contribute to the higher share of bites reported from cities [30]. It should be noted that these percentages reflect reported cases only and do not account for population size or differences in healthcare-seeking behavior, so they should be interpreted cautiously.

In the present study, most animal bites were caused by dogs (76.15%), followed by cats (20.79%). This pattern is consistent with findings from a national meta-analysis of over 250,000 bite cases [29], which reported dogs (81%) and then cats (13%) as the leading sources, although the proportions in our study differed slightly. Comparable species distributions have also been reported in Brazil, where dogs accounted for approximately 83–89% of reported aggressions and cats represented the second most common source, indicating a broadly similar pattern across middle‑income settings [31,32]. The importance of this finding lies in the fact that dogs remain the primary reservoir and the main target for rabies control in Iran, underscoring the need for sustained dog population management and vaccination programs [33]. At the same time, the relatively higher proportion of cat bites observed in our study highlights their potential role in transmission and suggests that cats should not be overlooked in educational efforts and post-exposure prevention strategies [34].

Lower limbs and hands were the most common bite sites in our study, a pattern that aligns with international findings from Bangladesh and Germany [35,36], as well as Brazilian data showing similar concentrations of injuries on the hands, feet and lower extremities [31,32]. High rates of wound irrigation (94.26%) and antiseptic use (76.96%) demonstrate strong adherence to WHO recommendations for immediate and thorough wound cleansing as a critical first step in rabies prevention [11]. The low rate of wound bandaging and closure aligns with guidelines that discourage sealing contaminated wounds to prevent infection [11,21].

Vaccination initiation was timely, with 96.37% of patients receiving the first dose within 48 hours, reflecting efficient early response consistent with WHO protocols emphasizing prompt PEP administration [11,21]. Adherence to subsequent doses decreased modestly by Day 14, a finding mirrored in Soleimanpour et al.[37], who identified age and rural residency as key predictors of PEP delay. These patterns underscore the need for active follow-up interventions, such as phone text reminders [38].

PrEP rates among high-risk occupational groups, including veterinarians (29.65%) and people working with non-domestic animals (10.46%), remained low, despite the national rabies control guideline and WHO recommendations emphasizing the importance of PrEP in these populations to reduce rabies risk [11,21]. These figures highlight a critical gap between guideline recommendations and real-world practice, suggesting that high-risk workers remain inadequately protected. Strengthening occupational PrEP uptake through systematic risk communication and targeted workplace interventions could therefore play a pivotal role in reducing rabies burden.

Among the 85,292 dogs assessed—based solely on patient self-report rather than verified vaccination records—overall rabies vaccination coverage was 35.3%. When classified by type, reported coverage was highest among hunting dogs (59.8%) and pet dogs (47.7%), while lower rates were observed in guard dogs (27.0%) and shepherd dogs (17.2%). According to the national rabies control guideline [21], all household (pet) dogs should be vaccinated against rabies; therefore, the observed 47.7% coverage among this group indicates a considerable gap. This highlights the need for enhanced owner education, improved access to veterinary services, and stricter enforcement of vaccination requirements. Nevertheless, since the information was not confirmed by vaccination cards or veterinary documentation, the accuracy of these rates may be limited due to recall bias or misclassification.

According to both the national rabies control guideline and the WHO recommendations, the appropriate management of suspected exposures is clearly stratified [11,21]. Category I exposures should only undergo wound washing, Category II exposures require both wound washing and rabies vaccination, while Category III exposures necessitate wound washing, full rabies vaccination, and the administration of RIG. When comparing these standards with the observed practices in our dataset, several important gaps in adherence become apparent.

Regarding wound washing, which is considered the cornerstone of rabies prevention across all exposure categories, was not documented in a proportion of cases. Specifically, it was absent in 13.52% of Category I, 3.63% of Category II, and 5.70% of Category III exposures. Since wound washing is expected in 100% of exposures regardless of classification, these findings indicate a significant deviation from established protocols. Possible explanations for this issue include insufficient awareness among healthcare providers, incomplete documentation in medical records, or misreporting during patient interviews. For example, if patients reported that they had already washed the wound themselves, the step may have been omitted from the medical records. Such omissions remain clinically important because inadequate wound care has consistently been linked with an increased risk of viral transmission.

Regarding rabies vaccination, an unexpectedly variable distribution of doses was observed across categories. The majority of patients in Categories II and III received the recommended three or more doses, although a fraction received fewer doses and in rare instances none at all. In Category I, where no vaccine should be given, 95% of patients nonetheless received between two and four doses, which suggests considerable over-treatment. A similar pattern of excessive vaccine usage has been documented in São Paulo, where large numbers of doses were administered despite not being indicated, reflecting systemic challenges in aligning clinical practice with national rabies guidelines [32]. In contrast, in Categories II and III, the subset of patients who received incomplete vaccination regimens may reflect several underlying factors such as patient loss to follow-up, discontinuation after confirmation of animal survival, provider misclassification, or errors in data entry. The recorded vaccine volumes further support potential data inconsistencies: while intradermal regimens typically require 0.1 ml or 0.2 ml per dose and intramuscular regimens 1 ml, a small proportion (1.39%) of intradermal entries were logged as receiving 1 ml on day 0 which is an implausible practice not aligned with WHO or national recommendations. This likely indicates data-entry errors where intramuscular administrations were inadvertently registered under the intradermal route. These deviations not only compromise the protective effect of prophylaxis but also indicate inefficiencies in the use of programmatic resources.

The administration of RIG aligns more closely with expectations in Category III, where 95.58% of cases received it. However, inappropriate use was identified in Categories I (6.13%) and II (4.66%), where RIG is not recommended. Comparable issues have been reported in the state of São Paulo, where RIG was frequently administered outside the national protocol, reflecting similar challenges in adherence to guideline‑based indications [32]. These findings suggest possible misclassification of exposure severity, confusion between pre- and PEP protocols, or provider overcaution. Brazilian studies likewise noted that errors in classifying exposure severity, together with inconsistencies in data entry, contributed to inappropriate RIG administration, indicating shared systemic vulnerabilities across surveillance settings [32]. Given the cost and limited availability of RIG, its inappropriate administration represents both a clinical and public health concern.

Although the overall post-exposure management aligns with recommendations, systematic deviations exist. These deviations are likely due to a combination of provider knowledge gaps, documentation errors, patient-related factors, and challenges in protocol adherence. Strengthening provider training, reinforcing standardized reporting, and implementing electronic decision-support tools could help close these gaps and optimize the use of available resources. Comparable deviations from protocol have been reported internationally. In China, a case-investigation of 10,971 rabies deaths (2006–2012) showed that only 9.6% underwent proper wound washing, 11.7% initiated vaccination, and just 3.9% of Category III exposures (234/5,927) received RIG, with frequent misclassification of Category I cases [39].

In our study, clear errors were identified in the way PrEP visits were recorded and managed. A total of 270 cases were incorrectly classified into exposure Categories I-III, even though this classification system is intended only for PEP after contact with a suspected rabid animal. Such mistakes suggest either limited awareness among providers about the distinction between PrEP and PEP, or possible misregistration of actual exposure cases under the PrEP category. However, given the structure of the national registry, it is not possible to determine whether these inconsistencies reflect data-entry errors, mislabeling of the visit reason, or overlap in documentation workflows. In addition, according to the national rabies guideline [21], PrEP should consist of a standardized two-dose schedule. However, only 82.4% of individuals received two doses, while many received three or even four doses, and a few had no vaccination recorded at all. These deviations point to a lack of familiarity with the correct protocol or, again, the possibility that some PEP cases were mistakenly recorded as PrEP. Other inappropriate practices were also documented, including administration of RIG, wound irrigation, antibiotics, and antiseptics in settings where they were not needed. All of these findings highlight weaknesses in training, documentation accuracy, and adherence to national guidelines. Targeted provider education, system-level alerts in electronic records, and regular audits may help reduce such errors and improve the rational use of resources. Similar patterns have been observed internationally. For example, a national survey in the United States showed that even in a well-resourced setting, many animal care workers had gaps in knowledge and engaged in inappropriate practices related to rabies PrEP and PEP [40].

The distribution of administered doses shows clear deviations from recommended regimens. According to both the Iranian national rabies prophylaxis guideline and the most recent WHO recommendations [21,41], the intramuscular schedule requires four 1.0 ml doses on days 0, 3, 7, and 14, while the intradermal regimen should consist of three doses of 0.1 ml per site (0.2 ml total) on the same schedule. In contrast, our data revealed that 1.10% of intradermal recipients had received four doses—a regimen not endorsed by current protocols and likely representing either administration or data entry errors. Moreover, the dose volumes among intradermal recipients were inconsistent with guidelines: while 96.12% appropriately received 0.2 ml on day 0, 2.49% were recorded as having received only 0.1 ml (suggesting single-site injection), and 1.39% as 1.0 ml, both of which are incompatible with the standard intradermal regimen. By contrast, all intramuscular recipients appropriately received 1.0 ml on day 0; however, only 48.08% followed the correct four-dose schedule, while 44.53% received three doses and 7.39% received two doses, again reflecting departures from protocol. Collectively, these inconsistencies highlight protocol violations or reporting inaccuracies, emphasizing the need for enhanced training and stricter adherence to both national and WHO recommendations.

According to the Iranian National Rabies Guidelines as well as the WHO recommendations [11,21], any exposure or bite by a bat should be classified as Category III. Such cases require thorough wound cleansing in addition to the administration of rabies vaccine and RIG. In our study, among 106 individuals exposed to bats, only 39 were correctly classified as Category III. A total of 41 patients received RIG, whereas 65 did not. Although all patients received the rabies vaccine, 9 of them completed only two doses instead of the full course. This misclassification may reflect a lack of awareness among healthcare providers and the public regarding the fact that bat exposures are inherently Category III. Moreover, the relatively low proportion of patients who received RIG highlights insufficient adherence to national and international guidelines. These findings underscore the need for further training and awareness-raising activities to ensure correct case classification and proper PEP in bat-related exposures.

Comparable findings have been reported internationally. In Croatia, nearly all bat exposures (92.9%) received appropriate PEP, including vaccine and RIG when indicated, demonstrating high adherence to guidelines [42]. In contrast, Choe et al.[43] described mass bat exposures in the United States and found wide variability in management, with 0–100% of exposed individuals receiving PEP depending on the event, largely due to the absence of standardized protocols. In Ontario, Canada, Middleton et al.[44] reviewed 166 bat-related cases in 2013 and observed that while 70% were appropriately classified as requiring PEP, 30% were unnecessarily treated despite no evidence of direct contact (bite, scratch, or saliva exposure), often because a bat was merely found in a room with a sleeping person. Together with our findings, where misclassification and underuse of RIG were common, these studies highlight that both under-treatment and over-treatment remain global challenges, and underscore the importance of consistent adherence to WHO recommendations for bat exposures.

This nationwide registry-based analysis highlights several important strengths observed within the documented management of rabies exposures in Iran, while also identifying areas that warrant further attention. The national bite registry demonstrates broad geographic coverage and captures a large volume of cases, enabling detailed epidemiological description of animal bite patterns and PEP practices. The structured and standardized nature of data collection facilitates assessment of documented protocol adherence and identification of potential gaps in clinical management and data completeness. Timely initiation of PEP, high rates of wound irrigation, and generally appropriate use of RIG in Category III exposures demonstrate reasonable alignment with both the National Rabies Control and Prevention Guideline and WHO recommendations. Collectively, these strengths highlight the system’s capacity to ensure rapid case management and provide a valuable evidence base for public health planning.

At the same time, some aspects could be improved. Documentation of wound washing was incomplete in a proportion of cases, inconsistencies were noted in vaccine dosing schedules, and occasional misclassification of exposure categories occurred. Similarly, certain challenges were observed in the management of PrEP cases and in the use of RIG for bat exposures. While these issues are not unique to Iran, they indicate opportunities for strengthening both practice and reporting within the surveillance framework, although detailed monthly or seasonal analyses could not be performed due to limitations in the available data.

In summary, findings from this nationwide registry-based analysis indicate that the national rabies registry in Iran serves as an important platform for documenting animal bite exposures and PEP practices. Among recorded attendees, timely initiation of post-exposure measures was frequently documented, although improvements in provider training, data accuracy, exposure classification, and adherence to recommended treatment schedules remain necessary. Broader rabies control strategies—such as increasing dog vaccination coverage, promoting PrEP among high-risk occupational groups, and expanding school-based education—should be informed by registry findings and aligned with documented epidemiological patterns. Strengthening data quality, reinforcing protocol adherence, and integrating registry-based evidence into prevention planning may support more efficient resource allocation and contribute to progress toward national and global rabies elimination goals.

## Conclusion

This nationwide registry-based analysis of more than half a million animal bite and prophylaxis records provides detailed insight into documented epidemiological patterns and PEP practices in Iran. Among registry-recorded attendees, timely initiation of PEP was frequently documented, reflecting substantial adherence to key national and WHO recommendations for rabies prevention. These findings highlight strengths in recorded case management while also identifying areas where improvements in documentation accuracy and protocol adherence are needed. Nonetheless, identified gaps in documentation, dosing accuracy, and case classification represent direct risks to patient safety, potentially leading to either life-threatening under-treatment or unnecessary resource expenditure. Linking surveillance more effectively with preventive measures is therefore essential not only for optimizing system performance but also for ensuring zero preventable human deaths. By addressing these gaps, Iran can further strengthen national rabies control and contribute meaningfully to the global effort toward rabies elimination. Ultimately, these actions will translate directly into enhanced public health security, safeguarding communities from this fatal yet entirely preventable disease.

## Supporting information

S1 Table Categories of Exposure to Suspected Rabid Animals. (DOCX)

S2 Table Wound Management and Antiseptic Application Following Animal Bite. (DOCX)

S3 Table Occupational Categories of Patients Included in the Study and Their Corresponding Job Types. (DOCX)

S4 Table Data completeness for key analytical variables (N = 507,404). (DOCX)
