# Supplementary material for: Nationwide registry-based analysis of rabies exposures in Iran: Epidemiological patterns and post-exposure prophylaxis adherence
Source: PLoS Negl Trop Dis. 2026 Sep 30;20(9):e0014751. doi: 10.1371/journal.pntd.0014751 (PMC13626360; doi:10.1371/journal.pntd.0014751)
Supplement: Supplementary file 1 [file pntd.0014751.s001.docx]

**Supplementary Table 1. Categories of Exposure to Suspected Rabid Animals**

| Exposure Category | Type of Contact or Bite | Recommended Medical Action | Notes |
| --- | --- | --- | --- |
| Category I | Touching or feeding animals; licking of intact skin | Wound irrigation with running water and soap | Non-invasive contact; minimal risk; no vaccine or RIG indicated |
| Category II | Minor scratches or abrasions without bleeding | Local wound care and immediate rabies vaccine | Injuries possibly exposed to contaminated saliva; vaccine required, RIG not indicated |
| Category III | Single or multiple deep bites, contamination of mucous membranes or open wounds with saliva, bat bites or scratches | Local wound management, immediate rabies vaccine, and RIG infiltration | High-risk sites (head, neck, hands, genitals); full post-exposure prophylaxis required, including for previously vaccinated immunocompromised individuals |

**Abbreviations:** **RIG:** Rabies Immunoglobulin - **PEP:** Post-Exposure Prophylaxis
