# Supplementary material for: Nationwide registry-based analysis of rabies exposures in Iran: Epidemiological patterns and post-exposure prophylaxis adherence
Source: PLoS Negl Trop Dis. 2026 Sep 30;20(9):e0014751. doi: 10.1371/journal.pntd.0014751 (PMC13626360; doi:10.1371/journal.pntd.0014751)
Supplement: Supplementary file 2 [file pntd.0014751.s002.docx]

**Supplementary Table 2. Wound Management and Antiseptic Application Following Animal Bite**

| Stage | Recommended Actions | Antiseptic / Virucidal Solutions |
| --- | --- | --- |
| Stage 1: Wound Irrigation | Vigorous irrigation with soap and running water for ≥15 min, under pressure | Soap/detergents – disrupt viral envelope; inherent virucidal properties |
| Stage 2: Antiseptic Application | Apply effective antiseptic solution after irrigation | Povidone–iodine 10%   Ethanol 70–90% Sodium hypochlorite 0.05–0.5% |
| Stage 3: Immunization (Vaccine & RIG) | For Categories II & III: immediate rabies vaccine; for Category III also local RIG infiltration | N/A |
| Stage 4: Referral & Follow-up | Hospital referral for complex wounds/allergic reactions; follow vaccination schedule | N/A |

**Abbreviations:** **RIG:** Rabies Immunoglobulin
