# Supplementary material for: Nationwide registry-based analysis of rabies exposures in Iran: Epidemiological patterns and post-exposure prophylaxis adherence
Source: PLoS Negl Trop Dis. 2026 Sep 30;20(9):e0014751. doi: 10.1371/journal.pntd.0014751 (PMC13626360; doi:10.1371/journal.pntd.0014751)
Supplement: Supplementary file 3 [file pntd.0014751.s003.docx]

**Supplementary Table 3. Occupational categories of patients included in the study and their corresponding job types**

| **Occupation** | **Description / Included Occupations** |
| --- | --- |
| Healthcare staff | Laboratory personnel and healthcare workers |
| Firefighters | Firefighters |
| Livestock farmers | Shepherds, farmers, and livestock owners |
| People working with non-domestic animals | Zoo staff, rangers, forest guards, animal care center staff, and hunters |
| Veterinarians | Veterinarians and veterinary technicians |
| Students | School and university students |
| Children | Children without occupation |
| Others | All other occupations not listed above |

All information on occupational categories is based on self-reported data from the individuals and recorded by the staff of the surveillance system. Precise definitions for certain categories, such as the exact age range for children, are not available; for example, a 10-year-old child may have been recorded as either a “child” or a “student.”
