# Supplementary material for: Nationwide registry-based analysis of rabies exposures in Iran: Epidemiological patterns and post-exposure prophylaxis adherence
Source: PLoS Negl Trop Dis. 2026 Sep 30;20(9):e0014751. doi: 10.1371/journal.pntd.0014751 (PMC13626360; doi:10.1371/journal.pntd.0014751)
Supplement: Supplementary file 4 [file pntd.0014751.s004.docx]

**Supplementary Table 4. Data completeness for key analytical variables (N = 507,404)**

| **Variable** | **Non-missing (n)** | **Missing (n)** | **% Missing** |
| --- | --- | --- | --- |
| Animal species | 502,480 | 4,924 | 0.97% |
| Animal vaccination history | 146,156 | 361,248 | 71.20%* |
| Antibody titer in animal | 185 | 507,219 | 99.96%** |
| Number of wounds | 469,379 | 38,025 | 7.49% |
| Serum administration | 507,343 | 61 | 0.01% |
| Delay in dose 0 | 504,811 | 2,593 | 0.51% |
| Delay in dose 3 | 405,229 | 102,175 | 20.13% |
| Delay in dose 7 | 341,760 | 165,644 | 32.64% |
| Delay in dose 14 | 34,166 | 473,238 | 93.27%*** |

* Animal vaccination history is available only for cases in which animal follow-up information was recorded.
** Antibody titers were assessed only in a small subset of vaccinated animals.
*** Day 14 vaccination applies only to individuals requiring the full multi-dose regimen according to protocol; therefore, missing values largely reflect cases in which this dose was not indicated.
